# Therapeutic antibody delivery: vector tools to boost efficacy and affordability

**DOI:** 10.3389/fimmu.2025.1714390

**Published:** 2026-01-07

**Authors:** Abhishek Chiyyeadu, Bushra Khan, Katrin Ehrhardt, Hildegard Büning, Michael Morgan, Axel Schambach

**Affiliations:** 1 Institute of Experimental Hematology, Hannover Medical School, Hannover, Germany; 2 REBIRTH Research Center for Translational Regenerative Medicine, Hannover Medical School, Hannover, Germany; 3 Division of Hematology/Oncology, Boston Children's Hospital; Department of Pediatric Oncology, Dana-Farber Cancer Institute; Harvard Medical School, Boston, MA, United States

**Keywords:** AAV vectors, antibody delivery, infectious diseases, lentiviral vectors, mRNA-LNP, plasma cell therapy

## Abstract

Antibody (Ab)-based therapeutics have become powerful tools across diverse disease areas, with advances in bioengineering giving rise to next−generation molecules designed to outperform conventional Abs. Yet, large-scale production and purification of such complex proteins remain costly and can restrict patient access. A promising alternative is to improve *in vivo* expression capabilities, which will reduce manufacturing burdens and improve safety and tolerability. Multiple gene delivery platforms - ranging from mRNA and viral vectors to engineered cell therapies - have matured considerably, as a direct result of years of clinical experience and growing regulatory confidence. The rapid deployment of mRNA vaccines against SARS-CoV-2, the clinical success of adeno-associated virus (AAV)- and lentiviral-based interventions, and the approval of chimeric antigen receptor (CAR)-T cell therapies highlight the potential of these technologies to transform how we deliver Ab therapeutics. While these approaches hold the promise to treat genetic aberrations in patients, they may also contribute considerably to advancing conventional Ab therapeutics against viral infections and other diseases through local persistence of the proteins. Looking forward, *in situ* expression may confer even more benefits for engineered Ab-like molecules, thereby compensating for possibly shorter half-lives and overcoming challenges in *in vitro* production and purification. Therefore, in this review, we critically evaluate how these established and emerging gene therapy platforms can be harnessed to expand access, and discuss possibilities to improve *in situ* availability through the choice of transient or stable expression systems to increase the efficacy of Abs and other therapeutic proteins. Furthermore, we explore the current landscape of technological advancements, identify key translational challenges, and project future directions for optimizing these approaches towards widely applicable clinical interventions.

## 1 Introduction

For several decades now, recombinant monoclonal Abs (mAbs) have revolutionized treatment strategies against a multitude of disorders, including but not limited to cancers, autoimmune disorders, and infectious diseases. The first U.S. Food & Drug Administration (FDA) approved mAb targeting CD3 on T cells, OKT3 (1986), was a murine mAb used to treat acute transplant rejection in patients who received renal allografts (1). Further developments in the field of Ab therapeutics led to the approval of mAbs with improved functionality to promote transplant engraftment, such as basiliximab (1998) and daclizumab (2016). These were either human-mouse chimeras or fully humanized Abs that significantly reduced the risks associated with species-related immunogenicity (2, 3). Besides the application of therapeutic Abs in transplantation medicine, breakthroughs such as the approval of ipilimumab (2011) against melanoma revolutionized the field of cancer immunotherapy. Ipilimumab blocks the inhibitory cytotoxic T-lymphocyte-associated protein 4 (CTLA-4) receptor on T cells, thereby boosting T cell activation and increasing cancer cell susceptibility to immune attack (4). As the field progressed, targeted chemotherapy in the form of Ab-drug conjugates (ADCs) made their way to the clinics. By effectively combining trastuzumab’s targeted binding of human epidermal growth factor receptor 2 (HER2) with the potent cytotoxic agent DM1, ado-trastuzumab emtansine (T-DM1) enables selective cancer cell killing while reducing systemic toxicity and side effects. T-DM1 was approved in 2013 for the treatment of solid tumors, specifically HER2-positive metastatic breast cancer (5). In addition, therapeutic Abs have proven indispensable in treating autoimmune disorders. Adalimumab, first approved by the FDA in 2002 for rheumatoid arthritis, has since demonstrated efficacy across multiple autoimmune conditions including plaque psoriasis and non-infectious uveitis among others (6). Another area where Abs have become more clinically important is the field of infectious diseases. Notable examples include palivizumab (2004) for respiratory syncytial virus (RSV), bezlotoxumab (2016) for Clostridium difficile infections, and ansuvimab (2020) for Ebola virus (7–10). The vitality of Abs in the context of infectious diseases was further emphasized by the approval of anti-SARS-CoV-2 Abs, such as sotrovimab and the combination of casirivimab and imdevimab, which significantly mitigated the severity and progression of the COVID-19 pandemic (11, 12). Despite subsequent advancements, clinical mAbs have continually presented limitations in terms of achievable efficacy, adverse events, requirement of large doses, and high compounding treatment costs. While research increasingly focuses on the molecular design of Abs, strategies for safe, effective, and economical delivery of Abs to patients remain areas with great scope for advancement (13–15). Emerging innovations in gene and cell therapy have substantially improved the safety and effectiveness of vector and autologous cell therapy platforms (16). The engineered cell therapy Kymriah, a CAR-T cell therapy against acute lymphoblastic leukemia (ALL), was approved relatively recently in 2017, marking a major milestone in the advancement in gene and cell therapies. This review focuses on the application of existing and novel gene delivery systems for targeted delivery of Abs to overcome the limitations of conventional Ab therapeutics. Viral and non-viral vectors, nanoparticles, and cell therapy are discussed with regard to gene delivery, safety and suitability for the treated disease types.

### 1.1 Engineering advances for Ab therapeutics

The sophistication of therapeutic Abs extends well beyond the mere humanization of such molecules, including innovations in their design and functional optimization. As a result, Abs are now clinically applied to address a variety of medical conditions. They can bind to molecules directly or indirectly block or activate cellular pathways that lead to inflammation, cell proliferation, cell death (apoptosis), or internalization of viruses. For example, ansuvimab blocks the cellular entry of Ebola virus by binding to the viral glycoprotein, while adalimumab inhibits tumor necrosis factor α (TNFα) to counteract systemic inflammation (17, 18). Following major breakthroughs in Ab technology, another key development was the demonstration by Biocca et al. (1990) that Abs can remain localized and functional within cells, pioneering the intrabody concept (19). Along these lines, the understanding of cellular uptake of human immunodeficiency virus-1 (HIV-1) Trans-Activator of Transcription (Tat) protein, later led to the identification of peptide sequences capable of mediating efficient intracellular delivery of proteins and other cargo molecules (20). The fusion of such peptides to intrabodies has enabled their delivery as recombinant proteins into the cytoplasm of targeted cells, to bring about their dedicated intracellular functions. Additionally, Abs can mediate the destruction of targeted cells and pathogens by engaging serum factors to trigger complement-dependent cytotoxicity (CDC) and recruiting immune cells for Ab-dependent cellular cytotoxicity/phagocytosis (ADCC/ADCP). Abs are also employed to deliver drugs by conjugation with small molecule chemotherapeutics or radioactive agents to generate ADCs and immunostimulatory Ab conjugates (ISACs) as modalities for precision medicine (21). Sacituzumab govitecan (IMMU-132) was one such promising ADC described in 2015 that demonstrated efficacy against several cancer types while partially conserving the ADCC capacity of its Ab component hRS7 (22, 23).

The inherent versatility of these molecules has enabled the production of Abs or Ab-like molecules by virtue of modifications to the antigen-binding fragment (Fab) and/or crystallizable fragment (Fc) for improved serum half-lives, inter-species testing, targeted localization, and generating multi-specific molecules with enhanced avidity and efficacy. The concept of bispecific Ab molecules was first introduced by Nisonoff and Rivers in 1961, laying the foundation for this innovative class of therapeutics. The seminal work by Schuurman et al. (1999) characterized the natural propensity of immunoglobulin (Ig) subclass G4 (IgG4) molecules to form bispecific Abs via ‘Fab-arm exchange’, thereby reinforcing confidence in bispecific reagents and guiding their rational design (24, 25). Decades later, in 2009, the field achieved a major milestone with market approval of the first bispecific Ab catumaxomab (Removab) against epithelial cell adhesion molecule (EpCAM) and CD3 markers to target malignant ascites that arise from epithelial carcinoma (26, 27).

**Figure 1:**
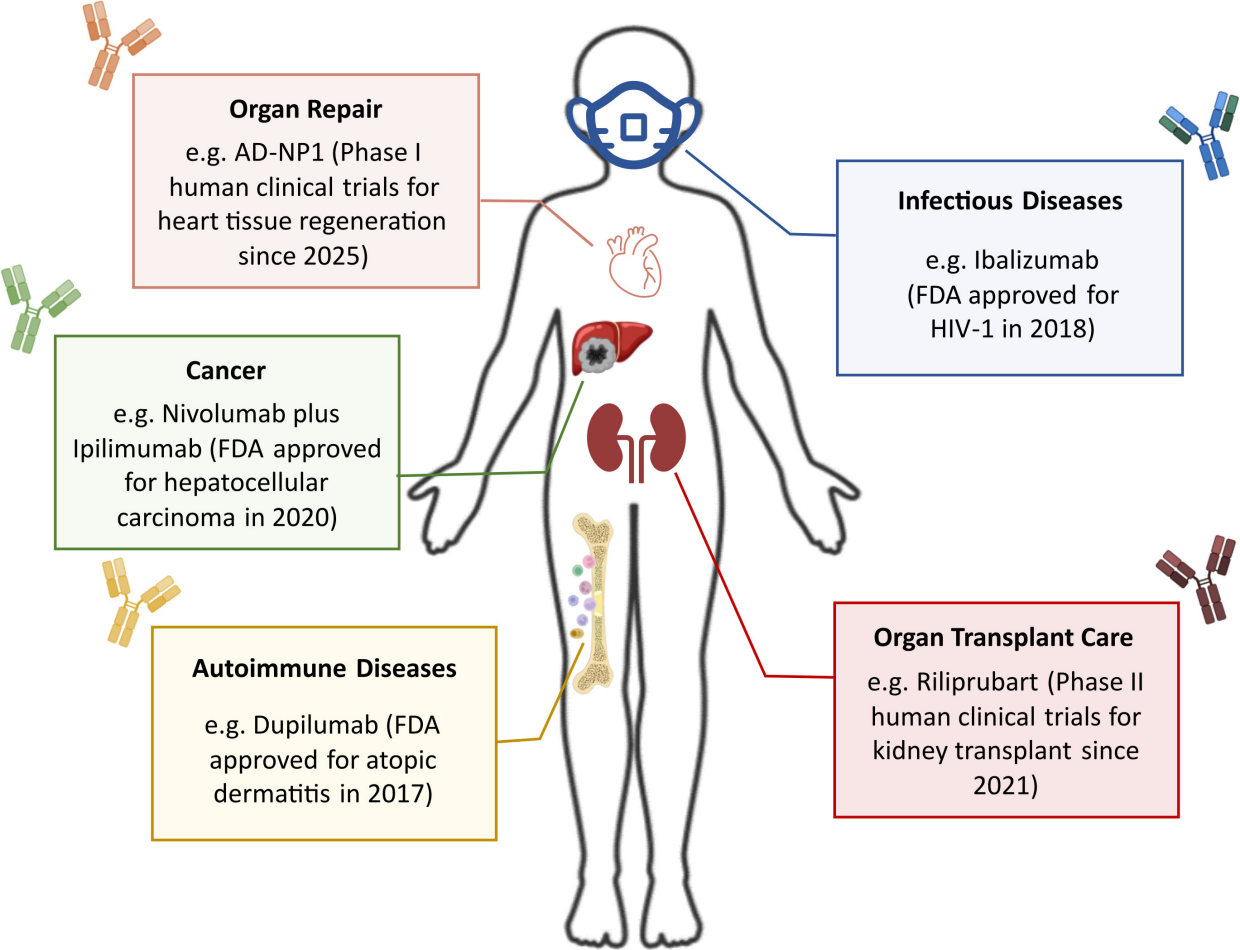
Applicability of antibody (Ab) therapeutics: For decades, Ab therapeutics have remained a versatile and promising approach to treat a broad spectrum of diseases and are supported by a favorable safety profile that continues to drive the development of new candidates. Initial efforts centered on transplantation medicine to improve recipient–donor compatibility. The greatest advances in the Ab field emerged in oncology, with subsequent progress in autoimmune disorders. Building on precedents such as convalescent plasma therapy, infectious diseases have also been on the radar for treatment using Abs (35). More recently, innovations have gravitated towards organ and tissue repair, offering the potential to reduce or even eliminate the need for transplantation (36, 37).

Owing to the global SARS-CoV-2 pandemic, there has been a notable rise in the clinical development of Abs against infectious diseases throughout the last four years. The pandemic revealed the vulnerability of our society to large-scale virus outbreaks. However, it also showcased the advances in modern medicine that led to the rapid development of novel antivirals in the form of small molecules and neutralizing Abs. In addition, research highlighted commonalities between SARS-CoV-2 and the earlier viruses, particularly of the coronavirus family, thereby facilitating repurposing of previously tested antivirals such as remdesivir and tocilizumab (12, 28–31). Ab-development and approval received significant thrust when five mAbs, namely bamlanivimab, etesevimab, casirivimab, imdevimab, and sotrovimab, were recommended for emergency use authorization (EUA) in clinics within two years into the pandemic. Furthermore, the pandemic spurred the generation of new bispecific Ab designs. These aimed to improve neutralization of forthcoming virus variants that were prone to evade the existing Ab treatments. In a recent publication, we demonstrated the potential of bispecific neutralizing Ab designs. Using state-of-the-art cell sorting and sequencing techniques, novel B cell-derived antigen-binding motifs were combined to generate a broadly neutralizing bispecific Ab, A7A9 TVB. Interestingly, A7A9 TVB potently neutralized several variants of SARS-CoV-2, some even better than the combination of parental mAbs A7 and A9 (32). These findings emphasize the importance of utilizing such bispecific designs to sustain our pandemic preparedness. Our research also underscores the potential to repurpose previously discontinued therapeutic mAbs and comprehensively upgrade the functionality of these particular antigen-binding motifs (32). Such repurposing initiatives can provide relatively low-cost solutions, bypassing the resource-intensive discovery and clinical translation phases of Ab development. Subsequent innovations include the generation of dual-function Ab-like fusion proteins that can inhibit productive viral infection while alleviating inflammation-related pathological manifestations. Wang et al. (2025) and Zhai et al. (2024) corroborated this idea by utilizing Ab-like molecules carrying mimetic peptides of mannan-binding lectins and angiotensin-converting enzyme 2 (ACE2) or a human interferon α2 (IFNα2) - trefoil factor 2 (TFF2) combination. These Ab-like molecules target the virus as well as treat lung inflammation during SARS-CoV-2 and influenza A infections, respectively (33, 34). In addition to mitigating future outbreaks by managing infectious diseases, the knowledge gained from these advancements is instrumental in building novel interventions for chronic fatal diseases such as cancers, autoimmune disorders, and organ failure (**Figure 1**).

### 1.2 Application-related challenges to Ab therapeutics

**Figure 2:**
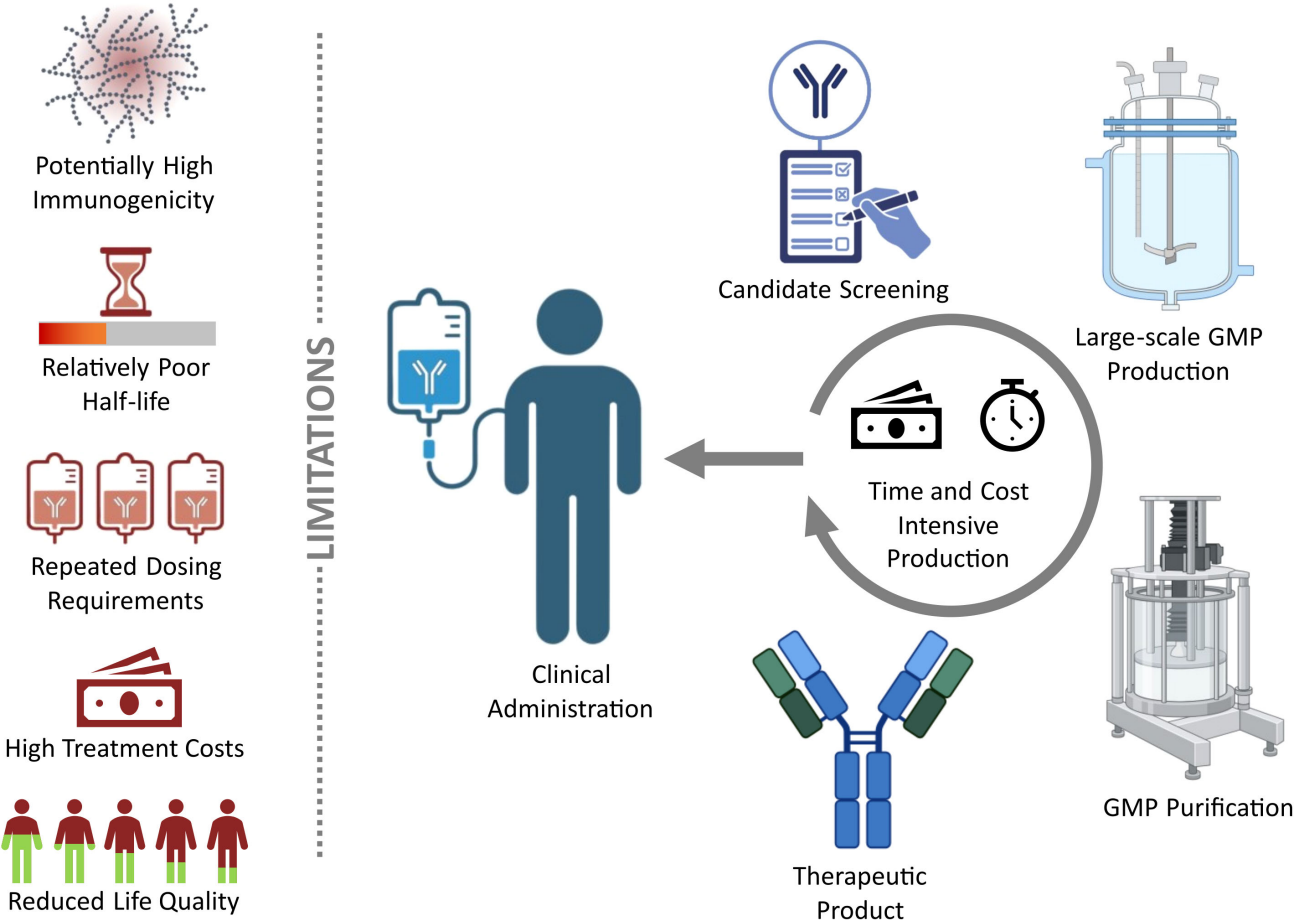
Challenges of conventional monoclonal antibody (mAb) therapies: The development of conventional mAb therapies requires up to $4.5 billion in research to reach clinical testing, with only 14–30% achieving regulatory approval after human trials (84, 85). Even under accelerated conditions, such as during the COVID−19 pandemic, the discovery of novel therapeutic Abs takes at least 5–12 months, in addition to an average of 85 months for clinical trial completion (52, 53). Furthermore, conventional mAb therapies repeatedly encounter challenges such as risk of immunogenic reactions, short half-life, frequent dosing, high treatment costs, and a compromised quality of life (56). GMP = Good manufacturing practices.

The expedited development of Ab therapies is of paramount importance in the context of infectious diseases and pandemic preparedness. However, the current gap in the treatment of refractory malignancies, autoimmune disorders, and organ tolerance or tissue repair remains a reminder of our failure to provide a long-term therapeutic solution to the patients in need (35–37). Despite the remarkable success of immunotherapies like immune checkpoint inhibitor (ICI) Abs, a large proportion of the patients encounter relapses or remain unresponsive to the treatment. With increasing use of ICIs, more patients develop acquired resistance, wherein the tumors evade or withstand treatment through intrinsic mechanisms such as the selection of resistant clones, genetic and/or epigenetic modifications, and loss of antigen presentation. Several meta-analyses reported that, of the patients treated with ICIs for advanced melanoma, 25 – 33% relapse within 11–14 months after treatment discontinuation (38–40). Similar to resistance in tumor cells, the phenomenon of acquired resistance can be observed in viruses as well. Rapid viral replication and accumulation of genomic mutations facilitate the selection of resistant clones under immune or mAb pressure. This dynamic was evident during the COVID-19 pandemic, as successive SARS-CoV-2 variants evolved with an increased resistance to existing therapeutic Abs (41, 42). Highly mutable viruses like HIV and influenza evade immune recognition through extensive antigenic variation achieved by frequent alterations in their surface proteins, thereby reducing the efficacy of Ab-mediated neutralization (43, 44). In addition, pathogens like *Neisseria gonorrhoeae* actively evade immune clearance through targeted disruption of Ab-mediated immune functions. Mechanistically, *N. gonorrhoeae* secretes IgA1 protease that specifically cleaves human IgA1 at its proline-rich hinge region. This impairs the ability of IgA1 to mediate an immune response, and promotes bacterial survival at mucosal sites (45, 46). Apart from the broad spectrum of diseases that challenge existing Ab treatments, conventional delivery methods further limit their effectiveness. Abs can be produced as recombinant proteins using transient or stable expression methods facilitated by gene delivery using chemical/physical transfection or viral vectors such as lentiviral vectors. However, recombinant proteins are currently more commonly produced in microbial, insect or mammalian cells using transfection methods, which tend to be rather inefficient (47, 48). Here, use of microbial cells to express human Abs may introduce risks such as incomplete or misfolding of amino acid chains or altered glycosylation, resulting in non- or dysfunctional Abs (49–51). Due to sequence-dependent expression variability, protein yields need to be individually optimized for each Ab candidate. The time-intensive screening and selection of therapeutic Abs is followed by additional stringent processes such as clinical-grade recombinant protein production and good manufacturing practices (GMP)-compliant purification of these proteins (**Figure 2**). Moreover, every batch of expressed protein requires thorough characterization to minimize the risk of non-specific effects caused by potential contaminants. This combination of inefficiency and rigorous quality control increases manufacturing and testing costs prior to clinical use (52, 53). The human immune system also poses significant hurdles, as it must recognize the therapeutic Abs as ‘self’ to avoid triggering a strong immunological response against the drug. This immunogenic potential is also greatly influenced by the source of the Ab, i.e., whether it was generated in cells from human or non-human species. In addition to immunogenicity concerns - mainly arising from suboptimal molecular design or non-self properties - the immune system may actively clear the Abs from the body. In addition, factors such as lack of neonatal Fc receptor (FcRn)-mediated recycling and cationization or increased isoelectric point (pI) of Abs may adversely affect half-life due to pronounced systemic clearance (54). Consequently, recombinant mAbs commonly have shorter half-lives of 6–32 days (**Table 1**) (55). To ensure therapeutic efficacy, frequent dosing regimens are often necessary, which increases the overall treatment cost. Despite ongoing efforts, circulating levels of these beneficial Abs remain transient, which may limit the extent of clinical improvements observed in patients (56). Furthermore, frequent and time-consuming mAb infusions require the patients to spend substantial time in the treatment centers impacting their quality of life (**Figure 2**) (57).

**Table 1:** Comparisons among delivery strategies.

Modality	Persistence	Repeat dosing	Cost per dose*	Clearance	Adverse events	Specialized patient management	References
Recombinant Ab	2–4 weeks	Yes, in most cases	$1000 - $5000	Fc-dependent metabolism/ADA/Liver	Mild injection reaction	No	(58–60)
mRNA	Weeks – months	Yes, in some cases	$80 - $170	Nucleases/Liver/Immune cells	Mild - moderate	No	(61–63)
AAV vector	Months - years	Yes, in some cases	$400K - $2M	ADA/Liver/Immune cells	Dose-dependent hepatotoxicity	Yes	(64–68)
Lentiviral vector	Years – lifetime	No	$250K - $500K	Phagocytes/Liver	Rare genotoxicity, inflammation	Yes	(67, 69, 70)
Cell therapy	Years – lifetime	No	$350K - $1M	Cell cycle/Immune cells	Cytokine storms, inflammation, autoimmune disorders	Yes	(68, 71–73)

The table compares the therapeutic persistence, repeatability of dosing, treatment cost, therapeutic clearance mechanisms, potential adverse events, and requirement of specialized patient care/management of different gene delivery methods. Each modality is associated with individual risks and benefits, and appropriate evaluation is essential prior to the choice of an intervention. ADA = anti-drug antibody, * = estimate.

Additional challenges are exemplified by the very first mAb therapy, OKT3, which aimed to improve the survival rate of kidney transplant patients by modulating and transiently depleting effector T cells. Statistics suggest that more than two million people suffer from kidney failure worldwide (74). Among other causes, a significant proportion of kidney injuries (nephropathies) are attributed to infections from viruses such as HIV, hepatitis B, hepatitis C, hantavirus, dengue virus and SARS-CoV-2, eventually leading to kidney failure (75–77). Nearly 40 years since approval of OKT3, about 20,000 patients continue to die within five years post-kidney transplant, mainly due to transplant rejection (74). Despite the initial success of OKT3 in preventing and treating acute rejection by binding and inactivating T cells, the incidence of immune reactions limited the long-term use of OKT3. On the one hand, up to 80% of patients demonstrated cytokine release syndrome (CRS) primarily due to an initial brief T cell activation induced by OKT3 cross-linking mediated by Fc gamma receptor (FcγR)-bearing cells, often necessitating treatment discontinuation. On the other hand, its murine origin triggered high immunogenicity that diminished therapeutic efficacy upon repeated administration. These limitations prompted the development of humanized and fully human mAbs with improved safety and reduced immunogenicity profiles as well as Fc silencing mutations to prevent binding to FcγRs and/or complement (78–81).

Although modern medicine has prolonged the life expectancy of humans, this success is balanced by a concomitant increase in disease occurrence in the geriatric population due to age-related inflammation (inflammaging) or weakening of overall immunity. These hurdles highlight the need for next-generation Abs to be incorporated into the patient’s immune system. Clinical experience with OKT3 provided fundamental insights that continue to guide the design of next-generation Ab therapeutics for transplantation and other immune-mediated conditions. Advances in gene/cell delivery systems can help modulate expression levels and duration to optimize and tailor Ab availability according to treatment requirements. Moreover, a weakened immune system directly correlates to an increased susceptibility to infectious diseases. Emergence of viral variants further challenges the clinically authorized therapeutic Abs by limiting their efficacies towards viral epitopes modified by acquired mutations. During the SARS-CoV-2 pandemic, mAbs developed through several months of research were withdrawn within a few months due to reduced affinities towards the glycoproteins of later variants such as Omicron (82). Such affinity losses can be compensated by elevating the mucosal concentration of the Abs to combat the virus in the target organs (83). Collectively, optimization of delivery strategies and advances in molecular engineering have greatly improved Ab-based treatment approaches.

## 2 Vector-mediated delivery of Ab therapeutics

Gene delivery platforms have enabled *in situ* expression of therapeutic genes for improved localization and thereby stronger effect of the encoded proteins in the affected organs. Tools like mRNA and adeno-associated virus (AAV) vectors offer requisite benefits suitable for diseases of transient nature, while limiting risks and side effects like genotoxicity that can be associated with protein overexpression from integrating vectors and genome editing technologies. In contrast, lentiviral vectors offer greater longitudinal effects with the potential for a lifelong cure, however with a greater risk of genotoxicity. Transplantation of *ex vivo*-modified allogenic or autologous B cells can also ensure durable expression of Abs and offer an attractive therapeutic approach for chronic ailments like cancer. These modalities for therapeutic Ab delivery are detailed below.

### 2.1 mRNA-mediated delivery

The discovery of mRNA as a tool to deliver genes revolutionized the field of vaccine development since the early 1990s. Wolf et al. (1990) first demonstrated the potential of mRNA-encapsulating liposomes as a tool to transfer reporter genes through intramuscular injections in mice (86). Since then, significant refinements have led to the development of commercially approved mRNA vaccines from Moderna and Pfizer-BioNTech. These vaccines were not only safe, but also immunized a large proportion of the global population against SARS-CoV-2, thereby providing timely relief during the pandemic (87). The inherent instability of mRNA was addressed by replacing natural nucleotides with chemically modified ones. Modified nucleotides such as pseudouridine enhance effective delivery without significant losses due to degradation and *in vivo* immunogenicity (88, 89). In addition, naked mRNA lacks the ability to cross the cell membrane and is defined to be strongly immunogenic. While chemically modified mRNA attempts to reduce the inherent immunogenicity, masking the mRNA from the extracellular immune system is a better alternative. Although encapsulating mRNA within liposomes enabled the transfer of transgenes, generating optimal liposomes and entrapping mRNA within them posed additional hurdles.

**Figure 3:**
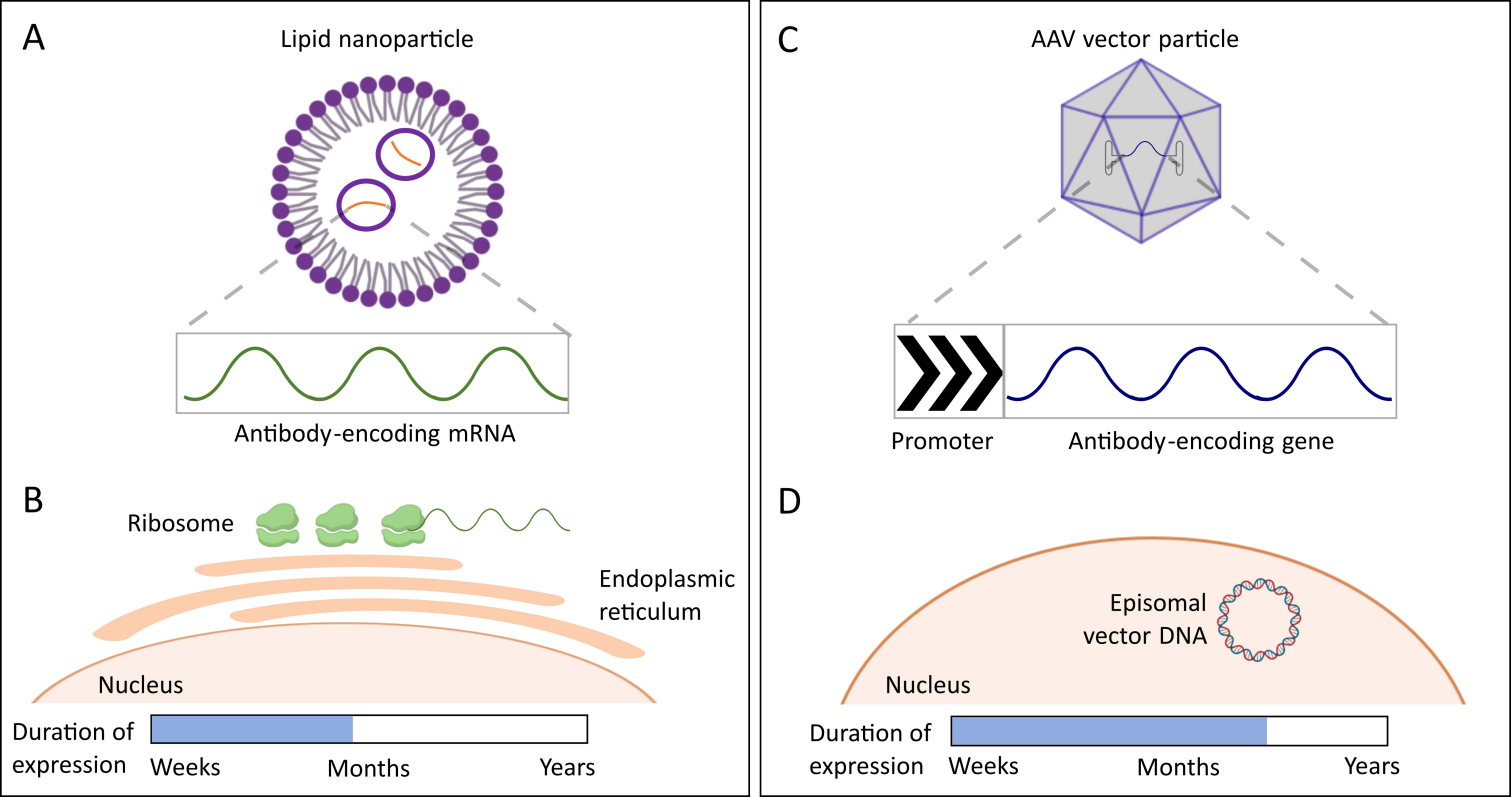
Transient delivery strategies: **(A)** The delivery of Abs using Ab-encoding mRNA encapsulated in lipid nanoparticles facilitates cellular uptake of the Ab-encoding gene. **(B)** The ready translation of such “self-made” Abs allows for continuous expression over a limited period of time, with better half-life profiles than recombinant proteins. **(C)** Adeno-associated virus (AAV) vectors are another elegant delivery tool to transfer Ab-encoding genes *in vivo*. **(D)** The genes are expressed from extra-chromosomally-located episomes, further prolonging the expression window of the “self-made” Abs up to several months or years, depending on the cell-type and rate of cell division, compared to a couple of weeks with mRNA-LNP-based strategies (**Table 1**). The reference to ‘duration of expression’ in the figure describes how long Abs generated by the delivery tools remain present and effective in the bloodstream. AAV = Adeno-associated virus.

The invention of lipid nanoparticles (LNPs) in 1991 as carriers for encapsulating nucleic acids such as mRNA marked a significant breakthrough in overcoming these hurdles (**Figure 3A**). Furthermore, LNPs have continually evolved through innovative engineering to address issues such as immune activation, early neutralization, stability, and targetability of these particles. While most advances in mRNA technology have emerged from the context of vaccine development, the same principles can be directly applied to Ab therapeutics. Herein, mRNA immunotherapies encode the therapeutic protein itself, enabling *in vivo* translation and secretion of functional Abs following transfection. This approach allows rapid and sustained Ab expression within host tissues, thereby improving the pharmacokinetics and dosing frequency. The advancements in the mRNA-LNP platform have opened the possibility of using these systems not only for passive immunization, but also for Ab therapy in patients. The current generation of LNP formulations for mRNA vaccines used in Onpattro (RNA interference therapeutic by Alnylam) and later in Spikevax (Moderna) and Comirnaty (Pfizer/BioNTech) has laid a strong foundation for the use of LNPs as gene delivery tools (90). In addition to flexible administration routes, research has enabled the engineering of LNPs for more specific and localized delivery of the encapsulated genes, thereby reducing losses due to hepatic clearance and increasing bioavailability in the targeted tissues (**Table 2**). It is now possible to develop peptide/protein decorated LNPs suitable for targeting neural retina and other organs like lungs (91, 92). Evidence from Radmand et al. (2024) and Qiu et al. (2022) suggests that organ-specific LNPs can also be generated by modulating the ratio of ionizable or cationic lipids in the formulation, thereby influencing the protein corona composition (93, 94). Therefore, targeted mRNA-LNP can avoid delivery to non-target organs and reduce undesirable systemic immunological reactions. Consequently, they have a better safety profile and lower dosage requirements compared to recombinantly produced protein formulations (**Table 1**). Pardi et al. (2017) and Deal et al. (2023) reported higher serum levels of HIV-1- and *Pseudomonas aeruginosa*-neutralizing Abs delivered through mRNA administration, in comparison to administration as recombinant protein. Furthermore, the translation-ready mRNA displayed rapid and durable protein expression in the study animals, which may translate to an increased overall efficacy required for a timely pathogen clearance (**Figure 3B**) (95, 96). These insights support the suitability of mRNA-LNP formulations as robust tools to deliver therapeutic Abs.

**Table 2:** mRNA-LNP compositions for targeted delivery.

Organ/Tissue	Key LNP composition	mRNA	Disease	References
Liver	Apolipoprotein E (ApoE)	Deficient or therapeutic protein (e.g. TTR)	Liver diseases	(99–101)
Spleen	1,2-dioleoyl-sn-glycero-3-phosphate (18PA)	Antigen-encoding, immune regulatory (e.g. CCR5 and PD-1)	Cancer immunotherapy	(100–102)
Heart	Anti-CD5 Ab	FAP-CAR	Cardiac fibrosis	(103)
Muscle	Newly synthesized ionizable lipid TCL053	Cas9 mRNA and sgRNA	Duchenne muscular dystrophy	(104)
Colon	Phosphatidic acid, monogalactosyldiacylglycerol, and digalactosyldiacylglycerol (5:2:3)	IL-22	Inflammatory bowel disease	(105)
Bone	Bisphosphonate (BP)	Bmp2	Bone or skeletal disorders	(106)
Blood (T cells)	Phospholipid PL1	OX40 costimulatory receptor	Melanoma	(107)
Neural retina	Peptide (MH42)	Cas9 mRNA and sgRNA	Inherited blindness	(91)
Lung	Sulfonium lipids (DHSEH and DOSEH)	CFTR	Cystic fibrosis	(92)

This table lists organs targeted by mRNA-lipid nanoparticles (LNPs) alongside the key LNP compositions and target disease. Bmp2, bone morphogenetic protein 2; CCR5, CC chemokine receptor type 5; FAP, fibroblast activation protein; IL-22, interleukin 22; OX40, tumor necrosis factor receptor superfamily member 4 (CD134 or TNFRSF4); PD-1, programmed cell death protein 1; TTR, transthyretin.

#### 2.1.1 Risk-mitigation

Ab delivery using mRNA has rapidly advanced, with meta-analytic data showing good tolerability and transient Ab expression reducing long-term safety concerns. Nevertheless, immune activation and biodistribution remain key considerations, and stringent immunogenicity testing is mandated in regulatory pathways to ensure safety in infectious disease contexts (97, 98).

### 2.2 AAV vector-mediated delivery

AAV vectors have been engineered for gene delivery since as early as 1984, taking advantage of the naturally non-pathogenic properties of AAV (108, 109). These vectors are small (~26 nm), non-enveloped particles that contain a single-stranded DNA genome (ssAAV) or self-complementary, double-stranded DNA genome (scAAV) (**Figure 3C**). Inverted terminal repeat (ITR) sequences at the 5’ and 3’ end of the viral genome serve as origin of replication and packaging signals (110). Their packaging capacity differs slightly between serotypes and is commonly limited to 5 kb. This constitutes a challenge for AAV vectors particularly when larger transgenes need to be delivered. To overcome this limitation, dual AAV vector approaches are used in which the promoter sequence and 5’ part of the gene are delivered by one vector and the 3’ part of the gene and poly A sequence are contributed by a second vector. Recombination of vector genomes, RNA transplicing or protein splicing strategies are followed to yield full-length transgene products following cell transduction (111). To overcome the limitations associated with the relatively limited packaging capacity of AAV vectors, research groups have also redirected their attention on engineering AAV vectors to express different Ab formats, such as full-length IgG, Fab, and single-chain variable fragments (scFv). Expression of these Ab formats is subject to its own advantages and limitations regarding *in vivo* delivery. While full-length IgG enables prolonged systemic Ab production, the large genome size and complex protein folding requirements restrict its widespread application. To address the packaging limitations, smaller Ab formats such as Fabs and scFvs are being increasingly utilized, due to shorter genomic sequences and improved tissue penetration, eventually resulting in enhanced and targeted gene delivery (112). Owing to technological advancements, AAVs have been successfully employed for *in vivo* Ab delivery (113). For instance, Gardener et al. (2015) used an AAV vector to deliver a CD4-Ig fusion molecule, which demonstrated potent neutralizing activity against simian–human immunodeficiency virus (SHIV) in two-year-old macaques (114). Yamazaki et al. (2018) demonstrated that anti-hemagglutinin Abs imparted protection against influenza virus in immunocompromised mice, when delivering the Ab-encoding gene using AAV vectors (115). In addition to the first generation of AAV vectors which use capsids from natural occurring serotypes, capsid engineered next generation AAV vectors with improved cell type selectivity and transduction efficacy are being explored (116, 117). A switch between capsids and thus a change in tropism and immunogenicity is simplified by the pseudopackaging technology as vector genomes flanked by ITRs of AAV serotype 2 (AAV2) can be packaged across the huge portfolio of available first and next generation AAV capsids. This strategy enables the use of the well-characterized AAV2 vector genome design to be packaged into the serotype capsid that best meets the requirement of a given application. Limberis et al. (2013) applied this strategy and used an AAV9 capsid to intranasally deliver F16, an anti-influenza Ab, that provided protection in both ferret and mouse infection models (118). AAV9 vectors are well-known as a delivery tool in Zolgensma, a gene therapy applied intravenously to transduce motor neurons in the brain in patients with spinal muscular atrophy (119). The blood-brain barrier crossing function of AAV9 vectors is thereby explored as a key factor of this serotype capsid. However, despite this feature, most AAV9 vectors accumulate in the liver. To overcome this limitation, capsid engineering has yielded novel AAV capsids that target the brain while being de-targeted from the liver. Such tools would ideally be suited to deliver Ab-encoding sequences to the brain following intravenous administration. In the central nervous system (CNS), AAV vectors are being explored for delivery of Abs targeting neurodegenerative disease biomarkers, such as amyloid beta in Alzheimer’s disease and alpha-synuclein in Parkinson’s disease (112, 120–124). While aducanumab is an approved human mAb that targets amyloid beta plaques in Alzheimer’s, experimental AAV-based gene therapies are under development for sustained CNS delivery of anti-amyloid Abs or binding proteins to enable long-term therapeutic expression. Regarding Parkinson’s disease, AAV-mediated overexpression of human alpha-synuclein in rodents recapitulates key Parkinson’s features, serving as a valuable model for development and evaluation of therapeutic strategies. Early-phase clinical development includes AAV vectors engineered to deliver Abs or Ab fragments aimed at sequestering or degrading pathological aggregates of alpha-synuclein to halt disease progression (112, 120–123). The use of engineered AAV serotypes with enhanced CNS tropism facilitates the transduction of neurons and glia, overcoming the blood-brain barrier challenges (112, 123, 125). The field of ophthalmology has been another prime area of clinical development for AAV vector-mediated gene delivery. One such example is ADVM-022 (Adverum Biotechnologies), a gene therapy product for the treatment of neovascular age-related macular degeneration (AMD), consisting of rAAV2 encoding aflibercept (an anti-VEGF protein). While clinical trials are currently underway to evaluate the safety and tolerability of therapy (NCT05536973), a single intravitreal injection has demonstrated significant clinical efficacy in Phase I clinical trials (NCT03748784) (112, 126–128). A distinguishing feature of AAV vector-mediated gene delivery is the persistence of vector transgene in the nucleus as episomes (**Figure 3D**). This enables sustained expression of the transgene product, in non-dividing or slowly-dividing cells, thereby outperforming mRNA-based approaches regarding the duration of therapeutic effects, while avoiding the genotoxic risks associated with viral vectors that integrate into the genome (**Table 1**) (129). Taken together, these characteristics underscore the advantages of AAV vectors and support their strong potential as delivery platforms for therapeutic Abs.

#### 2.2.1 Risk-mitigation

Vector-based delivery of Abs via AAV has shown promising long-term expression and protective efficacy. For example, AAV vectors encoding monoclonal Abs targeting SARS-CoV-2 achieved durable serum Ab levels with potent neutralization and protection in animal models, without inducing anti-drug Abs. Studies also demonstrate tissue-specific tropism influencing Ab distribution and immune engagement, highlighting safety and functional advantages in preclinical assessments. Meta-analyses confirm that although AAV delivery offers sustained Ab levels, regulatory approval for its clinical use requires monitoring of neutralizing Ab formation and insertional mutagenesis risks (98, 123, 130).

### 2.3 Lentiviral vector-mediated delivery

**Figure 4:**
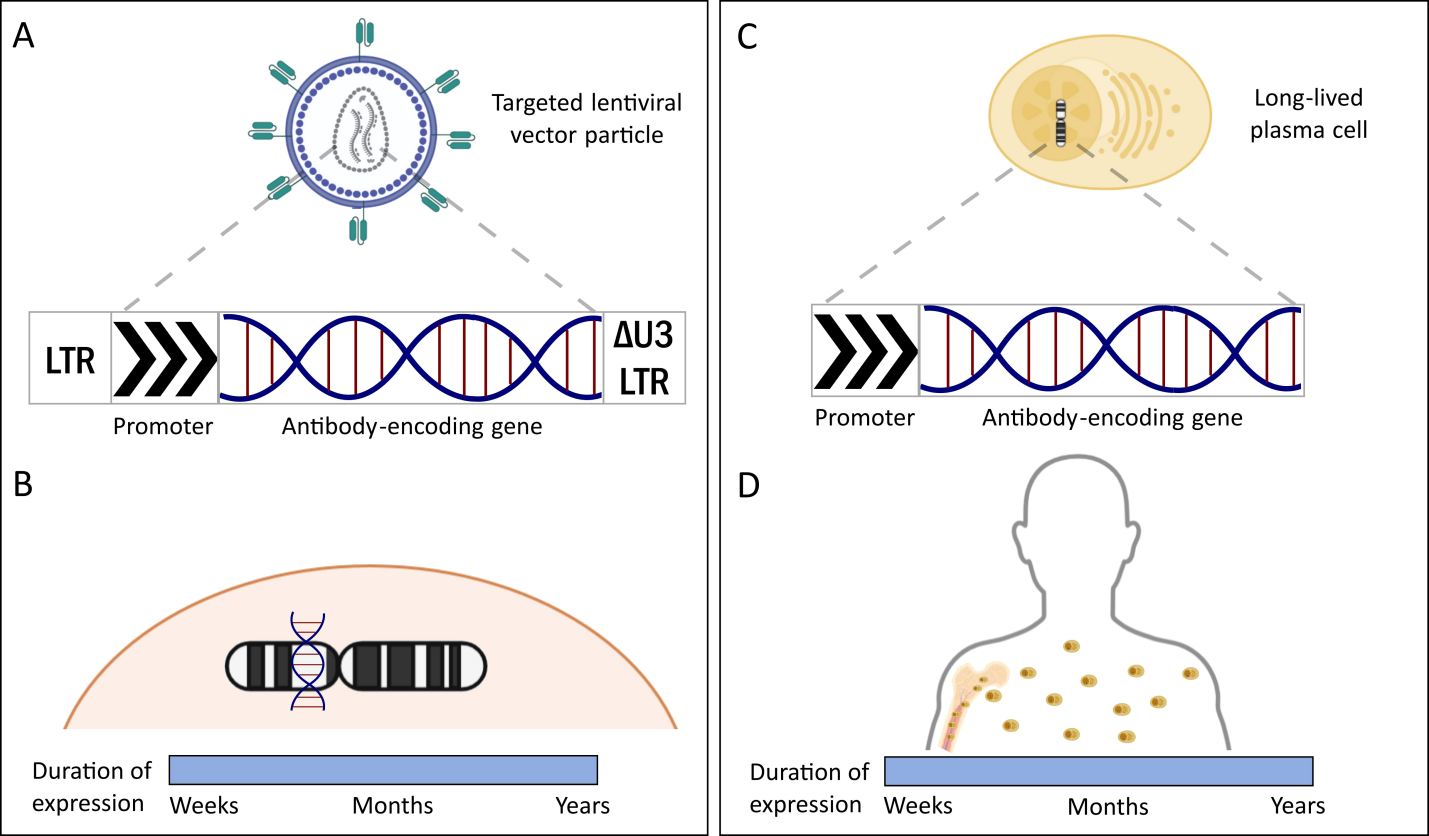
Stable delivery strategies: **(A)** Targeted lentiviral vector particles facilitate the delivery of Ab-encoding genes to specific cells, thereby limiting genotoxicity and transformation events upon *in vivo* administration. Shown here is the self-inactivating (SIN) (plasmid) configuration of a 3^rd^ generation lentiviral vector containing a deletion in the U3 region (ΔU3) of the 3’ LTR. **(B)** Lentiviral vectors integrate Ab-encoding genes into the host cell genome, ensuring persistent expression of the “self-made” Abs with a substantially longer serum half-life. **(C)** In contrast to *in vivo* applied lentiviral vectors, *ex vivo*-modified long-lived plasma cells can provide a more specific cell therapy alternative with steady expression of “self-made” Abs. The CD19+/-CD38hiCD138+ (long-lived plasma cell phenotype) cells are isolated from autologous sources, modified using vectors such as lentiviral vectors and expanded or differentiated in specialized culture medium conditions using cytokines such as B cell activating factor (BAFF) and APRIL (167). **(D)** Such an approach promises a higher serum presence of the therapeutic Abs by avoiding gene transfer to short-lived cells. The reference to ‘duration of expression’ in the figure describes how long Abs generated by the delivery tools remain present and effective in the bloodstream. Both lentiviral vector- and long-lived plasma cell-mediated Ab delivery lead to almost lifelong persistence of Ab levels in the serum. LTR = long terminal repeats derived from HIV-1.

Lentiviral vectors are gene delivery tools that utilize the viral machinery derived from HIV-1 and have a high nucleotide cargo capacity of up to 10 kb (131). Lentiviral vector particles carry two copies of vector genome inside a conical-shaped capsid and are generally pseudotyped with the glycoprotein of vesicular stomatitis virus (VSV-G), which imparts a broad tropism to these particles (**Figure 4A**) (132–134). This allows gene delivery to a wide array of cell types from diverse species. However, this desirable attribute often poses significant challenges to applying the lentiviral vectors directly *in vivo*, potentially leading to non-specific off-target modifications. Therefore, lentiviral vectors have primarily been used in clinics for *ex vivo* gene modification, thereby avoiding non-specific transduction *in vivo* (135). Due to their ability to stably integrate the transgene into the host cell genome, these vectors are seldom considered for the treatment of transient infection. Historically, instances where X-linked severe combined immunodeficiency (X-linked SCID) patients developed leukemia following treatment with LTR-driven gammaretroviral vectors due to insertional mutagenesis have generated substantial skepticism concerning the overall safety and use of integrating viral vectors (136). The current (3^rd^) generation of lentiviral vector particles, first described by the group of Trono and Naldini, has several safety optimizations over the earlier vector designs (137). This included the use of 4-plasmid split-packaging design, the deletion of accessory proteins, and the deletion of the 3’ U3 region in the so-called SIN (self-inactivating) LTR design. SIN vectors, especially with more physiological internal cellular promoters, have a reduced risk of insertional mutagenesis (138–141). However, concerns about the use of lentiviral vectors remain regardless of this enhancement. Since the transgenes are chromosomally integrated, these modifications can lead to permanent cellular dysfunction in post-mitotic cells—a cell type efficiently targeted by lentiviral vectors—highlighting ongoing needs to improve vector safety (**Table 1**) (142). Today, lentiviral vectors have also been successfully engineered to transiently deliver the restorative proteins or genes, similar to mRNA and AAV vectors, by inactivating the viral integrase enzyme. These non-integrating lentiviral vectors provide stable, but non-permanent, gene transfer and cannot cis-activate neighboring genes. Such vectors minimize the risk of insertional mutagenesis, making them a considerably safer alternative for gene therapy (143, 144).

To harness systematic gene delivery using lentiviral vectors and lower the risk of undesired cellular dysfunction, focused research efforts have enabled narrowing lentiviral vector tropism with precise selectivity to allow genetic modifications via *in vivo* administration of the vector particles (134). In addition to using glycoproteins from other viruses, advances to equip the lentiviral envelope with Abs or scFvs derived from Abs have demonstrated varying levels of success (134, 145–147). These modifications allow lentiviral vectors to selectively bind and deliver genes to desired cells while minimizing off-target effects and immunogenicity, with successful proof-of-concept demonstrated in preclinical studies. For example, bispecific Abs that bind both the lentiviral envelope and cell surface receptors have been successfully employed to enhance vector specificity in HER2-expressing cells, showcasing their potential for cancer gene therapy (148). Similarly, re-targeting lentiviral vectors with scFvs or other Ab domains has resulted in efficient and selective transduction of B cells *in vivo*, enabling functional gene delivery and therapeutic effects in lymphoma and hemophilia models (149, 150). Correspondingly, such a strategy to modify long-lived cells *in vivo* holds the potential to ensure incorporation of the Ab-expression cassette in the genome of the host at a safe (≤ 2) vector copy number (VCN), thereby providing a sustained serum supply of “self-made” Abs longitudinally (**Figure 4B**) (151–153). Prolonged expression of protective Abs achieved using lentiviral vectors has the potential to benefit against repeated exposures by transient pathogens as well as chronic ailments such as acquired immunodeficiency syndrome (AIDS) caused by HIV (**Table 1**). In addition, such a strategy imparts the ability to combat other latent viruses such as cytomegalovirus (CMV), lymphocytic choriomeningitis virus (LCMV), Epstein-Barr virus (EBV), human papillomavirus (HPV) and hepatitis B/C viruses (HBV/HCV) (154–156). Some of these latent viruses, such as EBV, HPV and HBV/HCV are known as oncogenic viruses and are implicated in causing malignancies. The persistence of Abs can prevent cancer occurrence in the infected patients, especially in cases of highly prevalent (90-95% of the global adult population) infections from viruses like EBV (157–159). Furthermore, malignancies in general can be targeted by sustained expression of anti-cancer Abs, such as the transformative immune checkpoint blockade Abs (160). Therefore, the *in vivo* delivery of therapeutic Abs using targeted lentiviral vectors holds great promise for the treatment of a multitude of chronic disorders.

#### 2.3.1 Risk-mitigation

Lentiviral vectors provide stable integration enabling durable Ab expression, showing efficacy in infectious disease models like HIV, with prolonged protection after a single administration. Safety concerns focus on the risk of genotoxicity related to transgene integration, which current mitigation approaches like suicide genes aim to address. Regulatory guidelines emphasize thorough analysis of integration sites and stringent vector quality control to minimize these risks (161–165).

### 2.4 Long-lived cell-mediated delivery

Long-lived plasma cells offer an attractive approach for sustained production of therapeutic Abs for longer than a decade (166). These cells originate from memory B cells, which can be isolated from autologous sources to reduce immunogenicity upon transplantation. *Ex vivo* differentiation protocols typically utilize cytokine cocktails to promote the maturation of engineered B cells into long-lived plasma cells capable of sustained Ab secretion (167). Their remarkable longevity and residence in specialized survival niches enable continuous, durable Ab secretion, making them ideal natural factories for long-lasting therapeutic Ab delivery with a single intervention (166, 168, 169). Cell therapies have demonstrated a considerable amount of success in cancer immunotherapies. The first documented cell therapy dates back to 1956, when E. Donnall Thomas performed a successful syngeneic bone marrow transplant operation to treat refractory leukemia (170). As of 2024, over 5,600 cell therapy clinical trials have been registered in the Cancer Research Institute’s Immuno-Oncology Intelligence database (171, 172). A major milestone in cell therapy was the FDA approval of the first anti-CD19 CAR-T cell therapy (2017), which supported the development of genetically modified cells as advanced therapy medicinal products (ATMPs) (173). In cancer treatment, significant advances have been made with the design of 4^th^ generation CARs (e.g. in TRUCKs). They combine CAR-mediated killing with inducible or constitutive expression of checkpoint blockade Abs to modulate the tumor microenvironment (174–177). Similar to *in vivo* genetic modifications by lentiviral vectors, *ex vivo* cell therapies using Ab-encoding gene-carrying long-lived cells enable longitudinal serum presence of therapeutic Abs against chronic ailments. Inserting the therapeutic antibody genes into “genomic safe harbors” (GSHs) can facilitate secure gene delivery (**Table 1**, **Figure 4C**). GSHs, such as AAVS1 and Rosa26, are loci that allow gene integration, while being non-disruptive to the host genome, thereby facilitating secure gene delivery and ensuring consistent Ab production (178–183). Modern technologies, including CRISPR-based gene editing and homologous recombination allow targeted introduction into these GSHs. Furthermore, pre-modified cells provide an added safety level of pre-selecting low-risk cells that *in vivo* modifications inherently lack. A 2022 study demonstrated that *ex vivo*-modified and differentiated plasma cells can engraft and secrete human Abs *in vivo* for over a year in the presence of human interleukin-6 (hIL-6) or human B cell activating factor (hBAFF) (184). Prior to this, Nahmad et al. (2020) proposed the idea of using modified B cells for long-term serum presence of broadly neutralizing Abs against HIV (185, 186). While cell therapies already provide an added risk mitigation through pre-characterization of the drug product, other advances to enhance safety include engineering suicide switches, such as caspase 9-based systems, which are well-characterized and used in ongoing clinical trials to eliminate therapeutic cells in case of serious adverse events (187, 188). While autologous transplantation mitigates host-versus-graft reactions and systemic immune hyperactivation, challenges such as high manufacturing complexity, cost, and the potential for non-specific immune reactions due to prolonged survival of engineered cells in the absence of target antigen create a bottleneck for the widespread application of cell therapies (182, 189, 190). With the potential to offer a lifelong benefit, cell therapies feature a favorable risk-to-benefit profile for longitudinal expression of protective “self-made” Abs with longer half-lives. These are especially valuable against chronic diseases that lead to patient mortality (**Table 1**, **Figure 4D**). Therefore, cell therapies offer a powerful and promising strategy to combat lethal viruses such as HIV, EBV and HBV/HCV. In conclusion, the convergence of cell engineering, precise gene-editing technologies, and advanced *ex vivo* differentiation methods enable the generation of long-lived plasma cells as ‘living drug factories’ for durable therapeutic Ab delivery. Ongoing refinements in target loci selection and safety controls continue to improve the translational prospects of cell therapies for chronic infectious and immune-related diseases.

#### 2.4.1 Risk-mitigation

Therapeutic plasma cell-based approaches enable continuous Ab secretion with immune tolerance benefits demonstrated in early studies with persistent Ab levels. Challenges include complex cell engineering and risk of non-specific immune reactions. Regulatory guidelines demand strict characterization of cell products and close monitoring for adverse effects post transplantation (98, 191–193).

## 3 Conclusion and future directions

Development of Abs and Ab-like molecules for therapeutic application continues to be a very active area of translational research. The remarkable versatility of these biologics to address diverse pathological conditions such as cancer, autoimmune disorders, and infectious diseases, underscores the medical need for continued refinement of molecular designs such as bispecifics, intrabodies, Ab-like fusion proteins, and ADCs. The advent of artificial intelligence has further strengthened *in silico* capabilities to design next-generation therapeutic molecules. Significant leaps in manufacturing processes for biologics, including vastly improved bioreactor designs and utilization of optimized protocols, promise a better commercial positioning of such interventions. Looking forward, the integration of these innovations with cutting-edge delivery systems will be crucial to unlock the complete potential of therapeutic Abs, and herald in a new era of accessible and affordable protein-based treatment strategies. Ultimately, success is dependent on the intrinsic efficacy of the antibody, but different delivery strategies and expression cassettes, including promoter strength, can clearly influence therapeutic efficacy, including expression duration, and safety.

### 3.1 Future directions

The rapid progress in designing novel therapeutic Abs or Ab-like molecules has outpaced the development of clinically translatable advances for improved delivery of these emerging biologics. Innovative administration routes such as inhalation and oral delivery are being progressively utilized in clinical trials for optimized local efficacy of therapeutic Abs. However, recombinant Ab production and purification still require significant resources, which further underscores the need to prioritize the development of efficient delivery systems for therapeutic Abs (194–196). While using vectors or cells to deliver Abs may not directly affect the absolute activity of the Ab molecule, they may greatly contribute to the bioavailability and the overall efficacy of the same therapeutic Ab compared to being delivered as recombinant protein. Gene delivery vectors aimed at introducing Ab-encoding genes into patients address two critical challenges: (1) “self-made” Abs produced endogenously tend to be less immunogenic than exogenous recombinant Abs, thereby reducing adverse events and minimizing therapeutic clearance; and (2) the Ab-encoding gene can be repeatedly translated *in vivo*, providing a sustained, “self-renewing” source of the therapeutic protein (**Table 1**). AAV vectors and mRNA delivery strategies are better suited for acute clinical manifestations that occur via pathogenic factors such as transient viral infections and require *ad hoc* treatment. Whereas lentiviral vectors and long-lived cell therapy may present a potentially favorable approach for life-threatening chronic manifestations, such as HIV infection-associated immunodeficiency. The delivery of Abs as protein formulations presents challenges including high production costs, complex manufacturing, limited shelf-life and stability, and frequent dosing requirements. Gene and cell delivery platforms have the potential to overcome these issues by enabling rapid, sustained, and targeted *in vivo* Ab expression. mRNA-LNP approaches offer transient and controllable Ab expression with rapid production and lower costs, but require optimized formulation to prevent immune activation and reduce accumulation in the liver. In contrast, AAV vectors enable long-lasting Ab expression and broad cell targeting. However, their limited cargo capacity restricts the delivery of large or bispecific Ab constructs, and their complex manufacturing processes contribute to higher costs. Delivery of large or complex Ab-encoding genes can be facilitated through the use of lentiviral vectors. While these vectors allow stable integration of the transgene, stringent safety measures must be applied to minimize the risks of potential genotoxicity. Cell-based therapies can furnish continuous Ab production with fewer immune side effects, but involve complex genome editing and costly manufacturing processes, with potential risks from long-term survival of engineered cells. In *in vitro* settings, the feasibility of virus-like particles for mRNA and protein transfer were shown. Galla et al. (2004 & 2008) and Voelkel et al. (2012) demonstrated a greater longevity of over 125 h of expressed proteins in the system using gene delivery approaches, as compared to 40 h using direct protein delivery (197–199). Both recombinant protein and gene/cell delivery approaches still face critical challenges and Ab efficacy can be compromised by issues like misassembly in complex bispecific or multivalent formats, immunogenicity, and manufacturing scalability. Thus, each modality offers distinct benefits and limitations that must be carefully assessed according to the therapeutic context and intended clinical use (**Table 3**).

**Table 3:** Ab delivery approaches.

Method	Advantages	Limitations
mRNA-LNP	Safe Low treatment cost Scalable production	Moderate immunogenicity Low durability (transient expression)
AAV vector	Safe Low immunogenicity, if engineered Moderate durability	High treatment cost Low scalability Pre-existing neutralizing antibodies
Lentiviral vector	Low immunogenicity Durable expression	Moderately safe High treatment cost Low scalability
Engineered plasma cells	Low immunogenicity Durable expression	Moderately safe High treatment cost Low scalability
Recombinant Ab	Safe Scalable production	Moderate immunogenicity High treatment cost Low durability

The table lists the advantages and limitations of different gene delivery methods.

The successful clinical administration of mRNA vaccines has notably bolstered confidence in mRNA-based therapeutics. Following its success against SARS-CoV-2, BioNTech has accelerated the clinical development of an mRNA-LNP platform to deliver bispecific Abs against advanced solid tumors, exemplified by the ongoing BNT142 trial by BioNTech (NCT05262530) (200, 201). Other promising avenues, such as self-replicating mRNA (srRNA), are currently explored to further increase the duration of therapeutic protein expression *in vivo* (202). In the realm of AAV vectors, more than 386 AAV-based gene therapy clinical trials have been registered (see also ASGCT quarterly report) that utilize local or systemic administration across various diseases (203). The preclinical development of Ab-encoding AAV vector particles is progressing rapidly, particularly for treating disorders in immune privileged organs, such as the brain and eyes. AAV-mediated delivery of Abs against targets such as the microtubule associated protein ‘tau’ and vascular endothelial growth factor A (VEGF-A) for the treatment of Alzheimer’s disease (AD) and age-related macular degeneration (AMD), respectively, is being evaluated in preclinical and clinical settings (127, 204). Furthermore, significant advancements in streamlining AAV vector manufacturing are underway, which could greatly improve the accessibility and scalability of these therapies (112, 205). Meanwhile, lentiviral vectors are extensively used for *ex vivo* cell engineering in patients with life-altering genetic disorders. Recent advances in envelope engineering and pseudotyping have remarkably enhanced cell-type specificity and safety of lentiviral vectors, paving the way for more Ab-delivering vectors to reach clinical use for chronic diseases (206). Notably, there have been considerable advances in engineered cell therapies, particularly with regard to improving the engraftment and safety of modified cells, as exemplified by the progress of CAR-T cell therapies (207, 208). As allogeneic transplantation and off-the-shelf cell therapy products evolve, we can anticipate the emergence of finely tuned cell therapies capable of controlled therapeutic Ab release for defined durations.

Despite their therapeutic promise, cell-based therapies remain considerably more costly and time-intensive to develop than conventional mAbs. The intricate manufacturing workflows, rigorous quality control, and stringent regulatory frameworks associated with engineered cell products necessitate substantial infrastructure investments, thereby constraining its scalability and clinical accessibility. Consequently, many companies are prioritizing next-generation Ab formats such as bispecifics and peptide-tagged mAbs with enhanced tissue targeting, including penetration of barriers like the blood-brain barrier (209). These platforms leverage existing manufacturing systems and clinical development pathways, offering a pragmatic equilibrium between innovation and feasibility that vector- and cell-based modalities have yet to achieve.

Recent breakthroughs in Ab engineering, combined with innovations in gene delivery vector technologies, present a critical opportunity to develop optimized, precise, and standardized strategies for delivery of therapeutic proteins. This integrated approach holds the potential to overcome persistent challenges in clinically used delivery methods, ultimately enhancing the efficacy and accessibility of protein-based therapies.
